# Gravisensation and modulation of gravitactic responses by other sensory cues in the monarch butterfly (*Danaus plexippus*)

**DOI:** 10.1242/jeb.245451

**Published:** 2023-11-07

**Authors:** Mitchell J. Kendzel, Adam F. Parlin, Patrick A. Guerra

**Affiliations:** Department of Biological Sciences, University of Cincinnati, Cincinnati, OH 45221, USA

**Keywords:** Gravisensation, Phototaxis, Magnetosensation, Oriented movement, Multimodal sensing, Sensory integration

## Abstract

Using the monarch butterfly (*Danaus plexippus*), we studied how animals can use cues from multiple sensory modalities for deriving directional information from their environment to display oriented movement. Our work focused on determining how monarchs use gravity as a cue for oriented movement and determined how cues from other sensory modalities, cues that by themselves also produce oriented movement (visual and magnetic directional cues), might modulate gravisensation. In two tests of gravisensation (movement in a vertical tube; righting behavior), we found that monarchs display negative gravitaxis only (movement opposite to the direction of gravity). Negative gravitaxis can be modulated by either visual (light) or magnetic field cues (inclination angle) that provide directional information. The modulation of gravity-mediated responses, however, depends on the relationship between cues when presented during trials, such as when cues are in accord or in conflict. For example, when light cues that elicit positive phototaxis conflicted with negative gravitaxis (light from below the monarch), monarch gravisensation was unaffected by directional light cues. We also found that the antennae play a role in gravity-mediated movement (righting), as, with antennae removed, monarch movement behavior was no longer the same as when the antennae were intact. Our results demonstrate that monarchs can use and integrate multiple, multimodal cues for oriented movement, but that the use of such cues can be hierarchical (that is, one cue dominant for movement), and the hierarchy of cues, and the responses towards them when found together, depends on the physical relationships between cues during movement.

## Teaser

**Summary:** Monarch butterflies can perceive and integrate multiple environmental cues from distinct sensory modalities (gravity, light, magnetic) to facilitate and guide oriented movement.

## INTRODUCTION

Animals can use different types of sensory stimuli derived from their external environment to facilitate oriented movement. For example, individuals can use multiple cues, each from a distinct sensory modality, to guide movement behavior. This use of multimodal cues is seen in many complex movement phenomena, ranging from long-distance migration (Muheim et al., 2006), mate recognition and localization (Griffith and Ejima, 2009), to predator avoidance (Stynoski and Noble, 2012). How organisms respond to multimodal environmental stimuli during movement can help identify the relative importance of sensory cues in triggering oriented responses, preferences and behavioral decisions. For instance, the responses of animals to multimodal cues presented together and in conflict (one cue elicits positive taxis whereas another elicits negative taxis) can indicate which cue is dominant or important in a behavioral context (Roth et al., 2016). Similarly, how animals respond to multimodal cues that can have modulating, complementary or reinforcing roles when sensed together can show how and when cues are integrated for eliciting behavior (Wessnitzer and Webb, 2006). Experiments that control or manipulate the presentation of multimodal cues when assaying animal movement phenomena can help determine the function of different cues, as well as illuminate potential mechanisms for how these cues are sensed, processed or integrated.

In this study, we used the monarch butterfly (*Danaus plexippus*) as a model to understand how animals can use and integrate multimodal sensory cues to produce oriented movement. Monarchs represent a useful study system for studying this phenomenon, as they can use environmental cues from different sensory modalities to robustly orient during their annual long-distance migration (Guerra, 2020; Guerra and Reppert, 2015). For example, eastern North American monarchs perform an iconic southwards migration during the autumn (fall season), as they leave their summer breeding grounds in southern Canada and northern USA to overwinter in central Mexico (Guerra, 2020; Reppert and de Roode, 2018). During migration, fall monarchs use compass mechanisms that derive directional information from environmental cues in different sensory modalities to maintain proper southwards orientation (Guerra, 2020). The dominant compass mechanism used by monarchs is a time-compensated sun compass, in which monarchs use visual cues such as the position of the sun in the sky and time of day information derived from their light-driven circadian clock to continuously fly southwards during the day (Froy et al., 2003; Mouritsen and Frost, 2002; Perez et al., 1997). On overcast days, when the position of the sun is occluded from view, monarchs will then use a backup magnetic compass, which uses the inclination angle of the Earth's magnetic field as an orientation cue that guides them to their overwintering sites (Guerra et al., 2014).

We first asked how monarchs might use and sense gravity for orientation during movement (walking) and body positioning (righting behavior), and then determined how gravity-mediated movement behavior is affected by cues from other sensory modalities. Although not well studied in monarchs and in Lepidoptera in general, gravity is a ubiquitous cue, and we hypothesized that gravity could play important roles during the oriented movement of monarchs in 3D space. For example, gravity can allow monarchs to unambiguously distinguish upwards from downwards in the vertical plane (i.e. *Z*-axis relative to the Earth's surface) throughout the day, a key piece of information during oriented movement relying on various compass mechanisms (e.g. when using an inclination-based magnetic compass; Wiltschko and Wiltschko, 1972; Guerra et al., 2014). Additionally, gravity cues can perform a key modulating role for monarchs when they are responding to directional cues in other modalities during oriented movement. Here, we specifically examined how gravity cues might interact with either light or magnetic cues. Overall, we found that monarchs do perform oriented movement using gravity cues (negative gravitaxis only) and that these responses to gravity cues can be modified by the presence of cues from other sensory modalities. We also show that gravity cues can be sensed by monarchs through different sensory pathways, with the antennae playing an important role during gravisensation.

## MATERIALS AND METHODS

### Animals

Adult monarch butterflies, *Danaus plexippus* (Linnaeus 1758), of both sexes were collected at the University of Cincinnati Center for Field Studies (Harrison, OH, USA: 39.28°N, −84.74°W) during the summer (2018, 2019) and autumn (2017–2019) field seasons. Monarchs were each placed in their own individual glassine envelope and housed in an incubator (Percival Model I-36LL, Perry, IA, USA). Summer monarchs were housed under summer-like conditions with a 14.5 h:9.5 h light:dark cycle (lights on: 06:00 h; lights off: 20:30 h), cycling temperatures (lights on: 29°C; lights off: 18°C) and constant relative humidity (70% RH). Fall monarchs were housed under autumn-like conditions with a 12 h:12 h light:dark cycle (lights on: 06:00 h; lights off: 18:00 h), cycling temperatures (lights on: 21°C; lights off: 12°C), and constant 70% relative humidity (RH). All butterflies were fed a 25% honey solution every third day. We complied with all relevant institutional and local animal welfare laws, guidelines, policies and regulatory standards during this study.

### Experimental light conditions

**Fig. 1.**
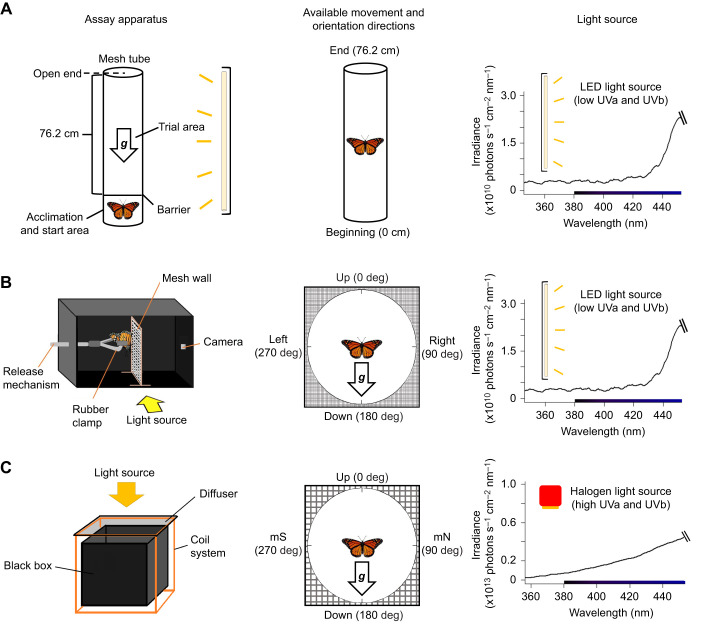
**Testing gravisensation in monarchs.** (A) Assay with vertical orientation (testing for negative gravitaxis). The mesh tube can be rotated upside-down or horizontally, and the light source can be positioned to the side, above or below the apparatus. The tube allows free movement either towards the end or towards the beginning of the apparatus. The LED light source (yellow bar) does not provide the necessary light input to activate monarch magnetosensation (Gegear et al., 2008; Guerra et al., 2014; Wan et al., 2021). The direction of the gravity vector is indicated by the arrow labeled ‘***g***’. (B) Righting response orientation assay. The opening to the black box allows control of the direction of the light cue, which could be on either side or below the plane of rotation (the mesh wall). The wall allows a monarch to orient in all possible orientations (360 deg). LED light source as in A. (C) Modified righting response orientation assay. Here, the black box (see B) is placed within a Helmholtz coil system, which generates an artificial magnetic field, allowing magnetic North and South to be aligned with the wall and axis of rotation, so magnetic South (mS) is on the left side and magnetic North (mN) is on the right side. With respect to the inclination angle of the presented magnetic field, equatorwards is on the left side and polewards is on the right side. The halogen light provides the necessary lighting conditions to activate monarch magnetosensation.

All trials for both experiments (see below for description of both the gravisensation assay and the righting response assay) were conducted in a darkroom with the specific light source used for the trial as the only source of illumination. As monarchs require exposure to ultraviolet-A/blue light wavelengths (380–420 nm; Gegear et al., 2008; Guerra et al., 2014; Wan et al., 2021) at a sufficient irradiance level (>10^11^ photons s^−1^ cm^−2^ nm^−1^; Helfrich-Förster et al., 2002; VanVickle-Chavez and Van Gelder, 2007) to activate their light-dependent magnetic sense, we used a light source that did not provide these lighting conditions when we wanted to prevent monarchs from using magnetic cues during trials. Here, we used a light (a single 108 cm long, 30 W white utility LED shop light; Commercial Electric, The Home Depot, Inc., Atlanta, GA, USA) that did not provide monarchs with this necessary light input (total irradiance of only 1.27×10^10^ photons s^−1^ cm^−2^ nm^−1^ between 380 and 420 nm; Fig. 1A,B, right). For trials that examined the use of magnetic cues by monarchs, we used a light source (250 W 4-in-1 Work light, LG Sourcing, Inc., N. Wilkesboro, NC, USA; Guerra et al., 2014) that did provide the necessary lighting conditions (total irradiance 7.02×10^13^ photons s^−1^ cm^−2^ nm^−1^ between 380 and 420 nm; Fig. 1C, right) that activates monarch magnetosensation. All trial light conditions were measured using a spectrometer (Ocean Optics Inc., Dunedin, FL, USA), equipped with an optic fiber (QP230-1-XSR, 235 μm; Ocean Optics Inc.) and cosine corrector (CC-3-UV-S; Ocean Optics Inc.), at the trial position of the butterfly within each apparatus. Spectrographs for analysis were generated using the ‘pavo’ R-package (Maia et al., 2013) to convert the values from radiance to photon flux.

### Gravitaxis assay

We first hypothesized that gravity is a cue that facilitates oriented movement in monarchs. To determine whether monarchs use gravity as a cue for directionality and in what manner, we characterized their responses to gravity cues using a tube assay that tests the role of gravisensation in movement behavior (Fig. 1A; Vang et al., 2012). The trial apparatus was modified from that used to measure gravitaxis in fruit flies (*Drosophila melanogaster*; Vang et al., 2012), and consisted of a cylindrical metal wire mesh tube (length: 90 cm) that included a 13.8 cm holding area in which a butterfly was placed at the start of a trial, and a 76.2 cm trial test section (Fig. 1A). The end opposite the holding area was open. The mesh tube had a diameter of 10 cm (approximately 1.5 times the body length of the butterflies tested), which provided sufficient space for butterflies to walk, turn and move in any direction within the tube during a trial. A thin wooden dowel (0.5 cm diameter) ran along the length of the mesh tube, serving both as an attachment point for the mesh cylinder and as more substrate for the organism to walk on. No movement bias was observed as a result of the presence of the dowel within the tube and the dowel provided no reference point in the vertical plane, as it ran equally the entire length of the tube. During all trials, the entire length of the mesh tube was illuminated equally by the 108 cm long shop light (held 61 cm away from the mesh tube), which did not provide the necessary light conditions for activating the magnetic sense of monarchs (see above). Further, trials were done in the center of a bare dark room to control for the use of landmarks, as per other gravitaxis studies (Armstrong et al., 2006; Vang et al., 2012).

For all trials assessing gravisensation, a monarch was removed from its individual glassine envelope and then placed in a mesh cage for 10 min outside the testing room to allow free movement before the trial. After this 10 min period, the monarch was then introduced into the mesh tube's holding area (Fig. 1A). The holding area was separated from the trial area of the mesh tube by a cardboard wall acting as a barrier, allowing the monarch to freely move within the holding area, while preventing it from accessing the trial area prior to the start of the trial. The wall was removed after 1 min, which then granted the monarch free access to the trial area and the entire tube. We then gave the monarch a maximum of 10 min to move in the mesh tube. For each trial, we measured the total absolute distance walked (in any direction) by the monarch, which served as an indicator of motivation for movement. We also measured the maximum distance that the monarch reached along the length of the trial area of the mesh tube, which was recorded as movement towards the open end of the tube relative to the starting point of the trial area. This measurement served as a proxy for directionality.

### Characterizing monarch gravitaxis

**Fig. 2.**
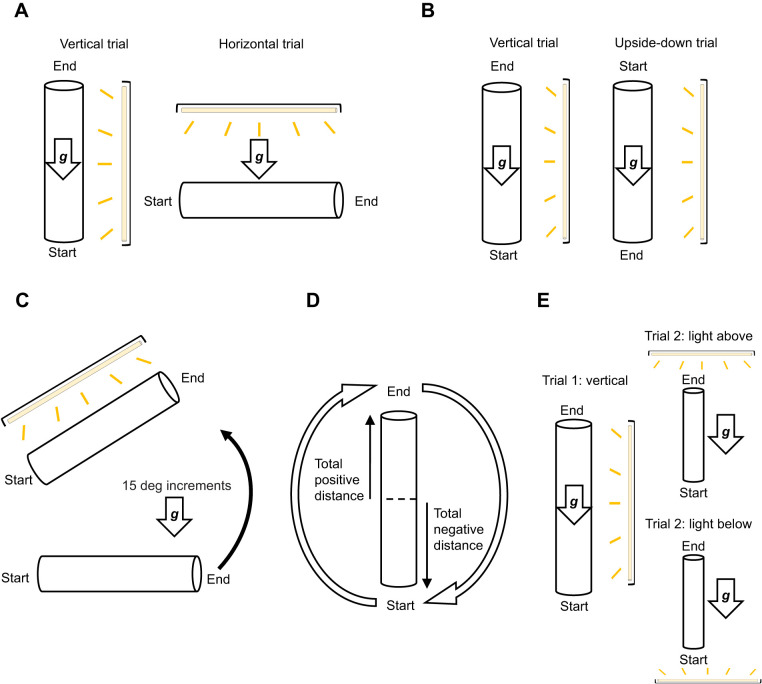
**Gravitaxis experiments.** (A) Negative gravitaxis (randomized trial order). (B) Positive gravitaxis (randomized trial order). (C) Inclinosensation (start at 0 deg). (D) Dynamic tube (flipped 3 times). (E) Phototaxis (trial 1 then trial 2 with light either above or below). Different subsets of monarchs were used in each experiment. Tube orientation in each trial is shown in relation to the gravity vector (arrow labeled ‘***g***’). For all trials, total distance walked within the apparatus and maximum distance reached toward the end were recorded.

To characterize how monarchs can use gravity as a sensory cue for facilitating oriented movement, we conducted five gravitaxis tests using our modified gravitaxis assay (Fig. 1A, left; Fig. 2A–E).

#### Negative gravitaxis

Migratory and non-migratory monarchs from autumn 2017, autumn 2018 and summer 2017 were tested in a set of two paired trials, with the order of the two trials randomized for each monarch: (1) the apparatus was perpendicular to the ground (treatment condition: vertical trial); (2) the apparatus was parallel with the ground (control conditions: horizontal trial) (Fig. 2A).

#### Positive gravitaxis

A different subset of autumn 2018 monarchs was tested in a set of two paired trials, with the order of the trials randomized for each monarch: (1) the apparatus was perpendicular to the ground with the opening at the top of the tube (same condition as in the vertical trial in the negative gravitaxis experiment to serve as a control); (2) the apparatus was perpendicular to the ground but flipped upside down (rotated 180 deg relative to the control condition) (Fig. 2B). In trial 1, each monarch started at the bottom of the apparatus, whereas in trial 2, each monarch started at the top.

#### Eliciting negative gravitaxis: inclinosensation

To test inclinosensation, the same apparatus that was used for both negative and positive gravitaxis experiments was systematically positioned at different angles relative to the horizontal (Fig. 2C). Using a subset of autumn 2019 monarchs, each monarch was tested in an initial trial that consisted of the mesh tube positioned parallel to the ground (horizontal position, 0 deg angle). After this initial trial, each monarch was then tested in a series of trials in which the apparatus was angled upward in 15 deg increments. Trials were continued until all monarchs displayed directed movement towards the open end of the tube. From these trials, we calculated the mean angle at which all monarchs began displaying negative gravitaxis. We also calculated the 95% confidence interval that contained all the observed angles at which monarchs exhibited negative gravitaxis.

#### Gravitactic behavior in a dynamic environment

In these trials (Fig. 2D), we tested summer and fall monarchs from 2019 in a modified mesh tube (length: 60 cm) that had no starting area or opening on either side, such that the two ends were identical during a trial. This modified mesh tube was illuminated using the same light source as in our other gravitaxis trials and was positioned 61 cm and parallel to the tube, to ensure equal illumination of the trial area. A trial consisted of placing a monarch in the modified mesh tube and allowing it to perform negative gravitaxis (walking up the tube). Once the monarch had reached the halfway point of the tube, we manually rotated the trial apparatus 180 deg at its midpoint, while the monarch was displaying negative gravitaxis. The total movement of the monarch either upwards or downwards after rotation of the tube was recorded until it reached one of the tube's ends. The total positive distance (walking up against gravity) and total negative distance (walking down with gravity) were measured from the midpoint of the apparatus after each rotation. Once the monarch reached one of the ends, the tube was then rotated again to reset the monarch's position back to the bottom of the apparatus. This process was repeated an additional two times once the monarch reached the midpoint, resulting in three total rotations during a given trial. The modified mesh tube was flipped three times to provide the monarch approximately the same total available distance to move as with the longer negative gravitaxis apparatus (see above). The tube was rotated at a pace slow enough to not startle the monarch during the trial, yet fast enough to complete the rotation before the monarch moved a significant distance (about a body length) in either direction (15 deg s^−1^).

#### Relationship of other sensory cues with gravitaxis

In this experiment (Fig. 2E), we tested monarchs in our mesh tube apparatus and manipulated the position of the light source such that it was perpendicular to the mesh tube (either above or below it), rather than parallel to it as in the previous experiments. To establish a baseline negative gravitaxis response in the monarchs used (control condition), we first tested autumn 2019 monarchs in trials in which the mesh tube was in a vertical position and the light was parallel to it. After demonstrating negative gravitaxis, monarchs were then randomly placed into either the light above or light below treatment groups. Their behavior in the second trial was compared with their baseline negative gravitaxis response.

### Righting response assay

Monarchs have a strong tendency to maintain a head-up position and are typically oriented in this manner in many key behavioral contexts (e.g. when nectaring, ovipositing or roosting), as it permits rapid takeoff and allows them to hang in position for long durations without expending much energy. This tendency to remain upward is seen across a wide range of animal species (e.g. locusts – Faisal and Matheson, 2001; turtles – Delmas et al., 2007; mice – El-Khodor et al., 2008; cockroaches – Ridgel and Ritzmann, 2005; honeybees – Hurst et al., 2014), with individuals immediately righting themselves (righting response or righting reflex) when upside down, head down or facing downwards. This self-righting behavior is important (Camhi, 1977; Frantsevich and Mokrushov, 1980), with animals moving from a poor position to a more advantageous one, as being inverted can leave them particularly vulnerable (for example, to predators). We therefore tested the righting response of monarchs and their potential use of gravity as a sensory cue that facilitates this behavior, by measuring how they can adjust their body orientation from a head-down to a head-up body position when on a vertical substrate in a novel orientation assay to test the role of gravisensation in body positioning (Fig. 1B).

We developed an assay to quantify how monarchs orient during righting behavior that consisted of a black cardboard box (dimensions: 68.6×33×36.8 cm; Fig. 1B, left) that had one side open during trials. The side of the box that was open (left, right or underneath the organism) depended on the specific trial that was being performed. This opening served as the illumination point and allowed the manipulation of where the light and visual cues were located during a given trial. A mesh wall (dimensions: 36.8×33 cm; Fig. 1B, center), was placed in the center of the black box, perpendicular to the ground. This wall served as the plane of movement for a monarch during a trial and allowed for full movement along the entire 360 deg of possible orientations in the vertical plane. A rubber clamp was threaded through the back wall, behind the monarch, and was used to manually hold the monarch in position via the wings, prior to the start of a trial. The rubber clamp could be opened and closed without the need to reach inside the box. Once the trial commenced, the rubber clamp was removed from behind the wall and the butterfly could move and rotate freely.

**Fig. 3.**
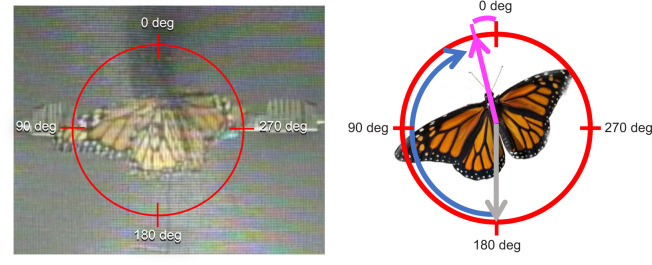
**Righting response trials.** Left: example of monarch righting behavior during righting response trials, with the red circle representing the total possible 360 deg orientation options during a trial. Orientations around the circle are mirrored given the position of the camera and measurements are taken with respect to the monarch's perspective (see Fig. 1B). Right: measurement of the monarch's orientation behavior during the righting response trial. The starting position (gray arrow) of the monarch during the trial was taken from the center of the body to the position of the head at the start of trials. The trial started with the monarch with its head pointing directly down and at 180 deg. During the trial, the monarch was free to move, and it moved clockwise from the starting point (blue arrow). The trial ended when the monarch stopped moving and maintained a final orientation direction (taken from the center of the body to where the head was pointing at the end of the trial); this was measured as the angle from 0 deg (purple line between 0 deg and the purple arrow). The red circle represents the total possible 360 deg orientation options during a trial (as on the left).

Prior to a trial, a monarch was placed in a mesh cage for 10 min outside the testing room to allow free movement before the trial. The monarch was then clamped within the box apparatus on the vertical mesh wall and held in place for 1 min in either a head pointed up position or a head pointed down position. After this 1 min holding period, the clamp was loosened, and the released monarch was given 5 min to select an orientation bearing while moving on the vertical mesh wall. Here, the orientation of the monarch was scored either as the direction it was walking in after it had walked one body length away from the starting position or the position it maintained at the end of the 5 min trial if the monarch was moving during the trial (e.g. rotating in place) but did not walk away from the starting position by at least one body length (see Fig. 3 for details). Orientation position was measured using a video recording system (I DVR-PRO, CCTV Camera Pros, Lantana, FL, USA) and analyzed using the ImageJ program (Schneider et al., 2012). The reference point for measurements for trials placed 0 deg as ‘up’ and 180 deg as ‘down’.

### Examining monarch gravisensation via the righting response

**Fig. 4.**
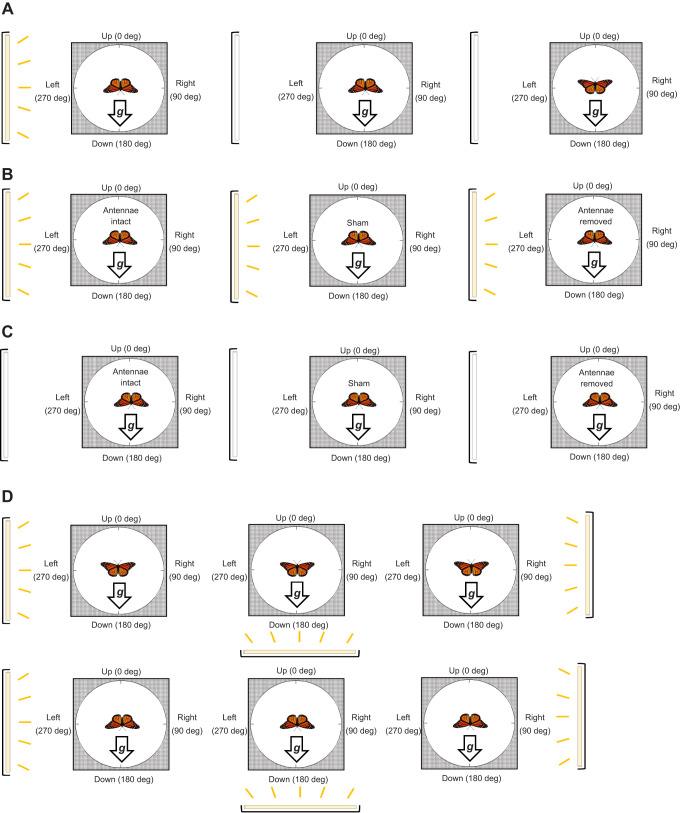
**Visualization of each righting response trial.** (A) Righting response in complete darkness. (B) Role of antennae. (C) Role of other gravisensors. (D) Gravity and light trials. For each subset of experiments in A–D, each monarch's first trial (baseline response) is represented on the left. For A, each monarch then did both trials (order of trials randomized) represented by the center and right panels. For B and C, after their first trial, monarchs were randomly assigned to the second trial in either the center or right panel. For D, each monarch went through all six trials (order randomized). The light source and its location during trials are indicated by a yellow bar when light was available or a white bar when light was absent. The direction of the gravity vector is indicated by the arrow labeled ‘***g***’.

We further characterized gravisensation in monarchs by examining their orientation behavior in three orientation tests (Fig. 4). In contrast to trials that examined gravisensation in our mesh tube assay in which monarchs were given either a binary choice (move up or down) or only a single option (move down only or move up only), the righting response assay provided monarchs with the ability to choose and orient in all 360 deg possible directions in the vertical plane during a trial.

#### Righting response in complete darkness

Monarchs from autumn and summer of 2019 were tested in three trials (Fig. 4A): (1) light on from the left (light that did not provide the necessary light input to activate monarch magnetosensation; see above), with the monarch's starting position head down; (2) in darkness, with the monarch's starting position head down; and (3) in darkness, with the monarch's starting position head up. Observations for trials done in complete darkness were made with an infrared camera system (HD-Q28, IR Bullet Camera, CCTV Camera Pros, Lantana, FL, USA). Prior to a trial, butterflies were held in an acclimation cage in darkness for 1 h.

#### Role of antennae

To identify potential candidate gravity sensors in monarchs, we examined the role of antenna in gravisensation by examining their role during righting behavior (Fig. 4B). As the antennae of monarchs function as multimodal sensory structures (Guerra and Reppert, 2015), and as the antennae have been found to be key structures for gravisensation in other insects (e.g. fruit flies, *D. melanogaster* – Armstrong et al., 2006; walking stick grasshoppers, *Carausius morosus* – Bässler, 1971), we hypothesized that the antennae might play a key role for gravisensation in monarchs. The antennae of lepidopterans contain Johnston's organs and Bohm's bristles (Sane et al., 2007), mechanosensory structures at the base of the antennae found to be important in assessing body positioning during lepidopteran flight. As the antennae of monarchs consist of a long, slender flagellar shaft with a bulb at the distal end that can act as a mass for gravity to act upon, the antennae might act as mechanosensors in a similar way for sensing gravity. Therefore, we predicted that without antennae, normal righting behavior would be disrupted in monarchs.

Monarchs from autumn and summer 2019 were tested in two trials: (1) with their antennae intact (baseline righting response) and (2) re-tested randomly after either their antennae were surgically ablated (treatment) or they had received a sham surgery (handling control). For all trials, the light position (light that did not provide the necessary light input to activate monarch magnetosensation; see above) was controlled and set to the left of the organism with their starting position facing head down. All surgeries (antennal ablation and sham) were conducted under a compound microscope. For antennal ablations, micro-forceps were used to hold an antenna in place, while micro-scissors were then used to remove the antenna in its entirety, where the joint connects with the head. This process was done to remove both antennae. Silicon grease (Danco, Irving, TX, USA) was used to seal the wound and prevent the organism from desiccating. Sham-operated monarchs received the same handling except their antennae were not removed. The second trial was conducted the day following surgery or sham-operation, giving animals a minimum of 24 h to recover from the stress of surgery and handling.

#### Other gravisensors

In this set of trials (Fig. 4C), we eliminated the potential role of the antennae in sensing gravity (as described above), and we eliminated the potential use of light cues for orientation by testing monarchs in darkness. Monarchs were first tested in the dark, with their antennae intact and with a head-down starting position (baseline righting response). After this trial, each monarch was then randomly chosen to either have their antennae removed (treatment) or receive a sham operation (handling control), prior to being re-tested. Surgeries and sham operations were conducted as above. Animals were allowed to recover from surgery/sham operation for 24 h then re-tested in the dark as in the first trial with a head-down starting position. Both fall and summer monarch butterflies from 2019 were tested.

### Sensory integration of orientation cues

#### Gravity, light and magnetic field cues during the righting response

In these trials (Fig. 4D), we tested monarchs at two different starting positions (either head up or head down) under three lighting conditions, for a total of six trials per monarch (with the order of the six trials randomized for each monarch), during which gravity cue-mediated righting behavior might be modulated by the presence of directional light cues. For all trials, light was presented consistent with the plane of rotation of monarchs in the apparatus. For two of the three trial conditions, light was positioned from one side only (the right side for one trial and the left side for the other) relative to the vertical plane in the apparatus. For the third trial condition, light was positioned from below the apparatus, perpendicular to the direction of the gravity vector. Monarchs from both autumn and summer of 2018 were used.

We additionally tested monarchs when directional light was inconsistent with the plane of rotation of monarchs. In these light conditions, light was presented from behind the monarch, effectively making light cues both perpendicular to the position of the monarch and with the plane of rotation during trials. We tested a different subset of autumn 2018 butterflies, with each monarch tested in three separate randomized trials: (1) light on the right side with a head-down starting position (baseline control for righting behavior); (2) light from behind with a head-down starting position (test for righting behavior); and (3) light from behind with a head-up starting position (comparison trial for different starting head positions).

Next, we tested fall migratory monarchs in a modified righting assay apparatus that was constructed to allow manipulation of the magnetic field that monarchs were exposed to during the trial (Fig. 1C). The righting response assay apparatus (dimensions, L×W×H: 41×31×21 cm) was nested within a Helmholtz coil system (two coils) that allowed us to generate artificial magnetic fields with different field parameter values for inclination angle and/or total field intensity (see Guerra et al., 2014). The horizontal coil allowed us to produce a field that aligned magnetic North and South with the monarch's axis of rotation and its testing position within the apparatus. Therefore, we made magnetic south (mS) on the left side and magnetic north (mN) on the right side of the monarch during trials (Fig. 1C, center). The vertical coil allowed us to manipulate the vertical component of the magnetic field such that we could alter the inclination angle of the field. Trial magnetic conditions were measured and calibrated using an Applied Physics Systems tri-axial fluxgate magnetometer (model 520A,; Mountain View, CA, USA) at the head position of the butterfly during trials. An opening above the apparatus allowed the illumination of the trial by our full-spectrum light source (which provided the necessary light input to activate the monarch magnetic sense; see above), with light running through a diffuser to provide diffuse, non-directional light (Guerra et al., 2014) (Fig. 1C, right).

We tested the righting response of monarchs under four artificial magnetic field conditions: (1) the ambient magnetic field at the testing location (University of Cincinnati, Cincinnati, OH, USA: 39.13°N, −84.51°W) based on the World Magnetic Model (WMM); (2) double the ambient field strength; (3) the ambient magnetic field but with the direction of the vertical component inverted 180 deg to shift the inclination angle from a positive to a negative value; and (4) the ambient field but with a 0 deg inclination angle. Prior to trials, monarchs were acclimated to each of the trial magnetic field conditions in a non-metallic mesh cage contained within a separate Helmholtz coil system, with the same light source, for 1 h in accordance with previous work (Guerra et al., 2014). We tested fall migratory monarchs (2019) in this experiment, with their starting position in trials head down.

### Statistical analyses

We assessed the displacement distances from each tube experiment (gravisensation apparatus) using a linear mixed-effect model (*lme()* function; https://cran.r-project.org/web/packages/lme4/index.html), which was compared against a null model using a likelihood ratio test, and then conducted a *post hoc* comparison using estimated marginal means (*emmeans()* function; https://cran.r-project.org/web/packages/emmeans/index.html). For orientation assays (righting response apparatus), significant mean orientations were calculated using either a Rayleigh's uniformity test or a *V*-test for non-parametric orientations. For multiple parametric pair-wise comparisons between trial groups, we used Hotelling's paired test (Zar, 1999) with adjusted *P*-values calculated using the false discovery rate (FDR) method (Benjamini and Hochberg, 1995). For antennae manipulation experiments (righting response apparatus), non-parametric analyses were used given the low sample sizes of these experiments and their departure from the Von Mises distribution. These tests included the Moore's paired sample second order test and a modified Rayleigh's test with a predicted *a priori* mean angle (also known as the *V*-test), given their increased statistical strength with low sample sizes (Landler et al., 2018). In these specific trials, we had an *a priori* prediction that monarchs would right themselves directly upwards (0 deg), given our results from the righting response experiments in which monarchs had intact antennae, which preceded experiments in which antennae were removed. Mean orientations were calculated using the R package ‘circular’ (https://CRAN.R-project.org/package=circular), adjusted *P*-values were calculated using ‘R’ and comparisons between trials were calculated using the program Oriana 4 (https://www.kovcomp.co.uk/oriana/; Kovach Computing Services, Pentraeth, Isle of Anglesey, UK).

## RESULTS

### Gravitaxis assays

**Fig. 5.**
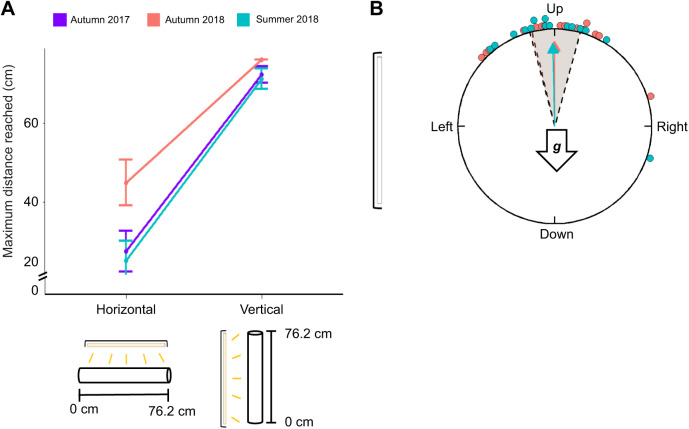
**Monarchs display negative gravitaxis.** (A) Monarchs walked farther toward the end of the apparatus in the vertical mesh tube, in line with negative gravitaxis behavior. Data are mean±s.e.m. distance walked. Lines connecting the data points represent the paired design of each year's experiment; monarchs performed both horizontal and vertical trials (autumn 2017: *n*=20; autumn 2018 and summer 2018: *n*=21). Below the graph is a simplified version of trial tube orientation and the single light's position (yellow bar) relative to the tube location. (B) In righting response trials, monarchs oriented upward (against gravity) when tested in complete darkness (white bar), which removed all visual cues. The results of individual trials are represented by circles; blue indicates monarchs from summer 2019 (*n*=15) and red indicates monarchs from autumn 2019 (*n*=15). Arrows are mean orientation direction with shaded areas indicating 95% confidence intervals. The arrow labeled ‘***g***’ indicates the gravity vector direction.

Monarchs moved significantly farther along the length of the mesh tube when in the vertical position and reached a greater distance relative to the starting point (bottom of the vertical tube), as compared with control conditions (tube in the horizontal position with monarchs starting at one end of the mesh tube). This pattern of directionality was seen with both summer and fall monarchs (likelihood ratio test: χ²=108.47, d.f.=3, *P*<0.001; Fig. 5A). To measure and compare the motivation and propensity to move by monarchs during trials, we used the total distance walked by each monarch in a trial as a proxy measurement. We found that the greater maximum distance reached by monarchs in vertical tube trials relative to horizontal tube trials (Fig. S1A) was not due to differences in the motivation to walk in the different conditions. The total distance walked by monarchs within a season (either summer- or autumn-captured monarchs) did not vary between vertical and horizontal tube orientations across all seasons (Fig. S1B). Moreover, in each season, monarchs had a similar propensity to walk during trials, as they moved a similar amount in the tube regardless of tube orientation (vertical or parallel) but reached a further distance along the tube relative to the starting point when the tube was in the vertical position.

When monarchs started at the top of the mesh tube (upside-down condition), they moved a significantly shorter distance along the length of the vertical tube and reached a significantly shorter distance relative to the starting point, as compared with trials when they started at the bottom (right-side-up control condition) (Wilcoxon signed-rank test: *n*=21, *V*=231, *P*<0.001; Fig. S1C). Furthermore, monarchs walked a greater total distance when they started the trial at the bottom of the tube, as opposed to when they started at the top (Wilcoxon signed-rank test: *n*=21, *V*=231, *P*<0.001; Fig. S1D).

Negative gravitactic behavior was elicited as monarchs shifted from random, back-and-forth walking to oriented movement along the length of the tube, after the angle of the mesh tube was tilted greater than 60 deg relative to the horizontal in trials. Here, when the angle position of the tube increased, the total walking distance performed by monarchs in the tube reached its maximum at 60 deg (likelihood ratio test: *n*=10, χ²=23.38, d.f.=4, *P*<0.001; Fig. S2A). Moreover, the total distance walked by monarchs significantly decreased, with less back-and-forth movement observed, as the angle of the mesh tube was increased across all tube angle positions (likelihood ratio test: *n*=10, χ²=18.31, d.f.=4, *P*<0.01; Fig. S2B).

In the dynamic environment trials, when the tube was flipped during gravitactic behavior, summer and fall monarchs had similar total negative distance (walking down) and total positive distance (walking up) traveled within each measurement. For each measurement, however, total negative distance values were significantly lower than total positive values (likelihood ratio test: χ²=84.36, d.f.=2, *P*<0.001; Fig. S3).

When we tested monarchs with directional light cues and gravity simultaneously, we found no differences between the placement of the light above, below or parallel to the mesh tube on the total distance (likelihood ratio test: χ²=7.11, d.f.=3, *P*=0.07) or maximum distance walked by monarchs (χ²=5.62, d.f.=3, *P*=0.13; Fig. S4). These results indicate that the co-occurrence of light cues, such as directional light cues from either above or below the tube during trials, had no effect on negative gravitaxis in monarchs.

### Righting response assays

Across all experiments, monarchs significantly oriented their movement and positioning, demonstrating that they can derive directional information for oriented movement across the different sensory contexts that we presented them with (Tables S1 and S2). In addition, the starting position of monarchs, either head down or head up, did not affect their final orientation in any experiments (Hotelling's paired test for head up versus head down: *P*>0.05 for all trials; Table S1), as their final orientation was equivalent when starting either head up or head down. This result indicates that monarchs possess a preferred orientation (head up) that is unaffected by their starting position.

In complete darkness, monarchs were able to right themselves and orient upward and against the direction of gravity (Fig. 5B). Our results demonstrate that gravity is sufficient for proper righting behavior, even in the absence of light or magnetic field cues, as these cues were unavailable in complete darkness.

**Fig. 6.**
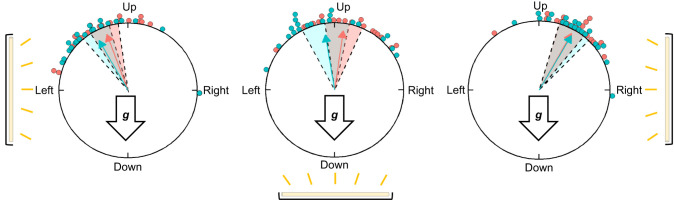
**Effect of light position relative to the direction of gravity on vertical body orientation during righting behavior.** The location of the light source deflects monarchs from their upwards orientation towards the position of the light source when light is perpendicular to the direction of the gravity vector (left and right panels). When the light (which elicits positive phototaxis) is below the apparatus and thus induces sensory conflict with negative gravitaxis, there is no observed change in the upwards orientation of monarchs, suggesting that gravity is the dominant environmental cue (center). For all panels, blue circles indicate summer 2018 monarchs (*n*=21) and red circles indicate autumn 2018 monarchs (*n*=21); colored arrows represent mean orientations for the trial group with shaded areas indicating the 95% confidence intervals. The yellow bar represents the single light source's location for each trial. Arrows labeled ‘***g***’ indicate the gravity vector direction.

In experiments in which monarchs were presented with gravity cues and directional cues from another sensory modality simultaneously (either light or magnetic cues), we found that these cues were able to modulate righting behavior. When we presented directional light cues in tandem with gravity cues, but not magnetic cues as the light source used did not provide the necessary light input to activate monarch magnetosensation, monarchs moved to the head up and facing upward position, but their final orientation was then additionally shifted towards the position of the light source depending on where light was coming from. Directional light modulated monarch orientation when it was positioned on either the left or right (Hotelling's paired test: *P*<0.001 for all; Fig. 6), causing them to shift their orientation toward the light when in the head-up position (Fig. 6, left and right panels). When light was directly below the butterfly, so that light and gravity cues would place negative gravitaxis and positive phototaxis in direct conflict, monarchs oriented upward as normal, with no monarch ever orienting and facing downwards (Fig. 6, center panel). When light was presented behind the butterfly and not on the plane of rotation, monarchs oriented upward as normal, demonstrating no effect of directional light coming from this position (Table S1). Here, we observed that the final orientations of monarchs were not similarly deflected from upwards when the light was placed behind them compared with when it was placed laterally to the butterfly (Hotelling's paired test; light on right versus light behind: *n*=10, *f*=7.6, *P*=0.01). All these trends were seen in both fall and summer monarchs (Table S1).

**Fig. 7.**
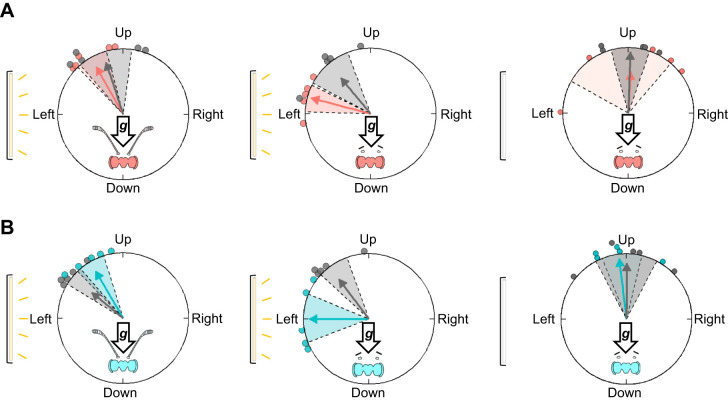
**Role of antennae in gravisensation.** For both (A) autumn 2019 (*n*=5) and (B) summer 2019 monarchs (*n*=5), antennae were important for proper upwards orientation during righting behavior. Results of sham trials with the light on (left) are consistent with our light trials where upwards orientation was shifted towards the light source. When antennae were removed (center), monarchs oriented directly towards the light source instead of upwards. When both the light and antennae were removed (right), monarchs oriented directly upwards, suggesting the presence of a secondary gravity-sensing mechanism. For all panels, the colored and gray arrows represent the mean orientation of monarchs, shaded areas are 95% confidence intervals, and circles represents individual post-sham operation or post-operation trial results (red: autumn 2019 monarchs; blue: summer 2019 monarchs). Gray circles represent pre-trial baseline responses for each monarch. Arrows marked ‘***g***’ indicate the gravity vector direction and below this arrow is a simplified monarch head and antennae; for trials with antennae removed, the monarch head lacks antennae in the figure. The light source used and its location during trials are depicted using a yellow bar for light available or a white bar when light was absent.

We found that the antennae play a key role in righting behavior (Table S2). Antennae were important for proper upward orientation in trials with the light on, as monarchs shifted their orientation toward the light source when they had their antennae removed (Moore's paired test pre- versus post-surgery; autumn 2019: *R*′=1.33, *P*<0.001; summer 2019: *R*′=1.32, *P*<0.001). In contrast to control trials (baseline trials with antennae intact), antennae-less monarchs oriented directly towards the light source instead of upwards and against the downward direction of the gravity vector (Fig. 7, center panels). Fall monarchs in sham trials had similar upward orientations between their baseline and post-sham operation trial (Moore's paired test: *n*=5, *R*′=0.62, 0.5>*P*>0.1; Fig. 7A, left). For summer monarchs, sham trial orientations were different from baseline trials (Moore's paired test; *n*=5, *R*′=1.32, *P*<0.001; Fig. 7B, left), but there was significant overlap in the orientations between the two conditions, and post-sham operation trial orientations were consistent with previous results from trials in complete darkness (see above) and when the light was on the left (see below).

We found evidence for an additional mechanism for gravisensation in monarchs independent of the antennae, as we found that monarchs righted themselves normally when their antennae were removed (Table S2). This secondary mechanism for gravisensation did not rely on monarchs having access to light and magnetic cues for orientation. Both summer and fall monarchs rotated toward the head-up, facing upwards position with and without antennae in complete darkness (Moore's paired test: autumn 2019: *n*=5, *R*′=0.62, 0.5>*P*>0.1; summer 2019: *n*=5, *R*′=0.20, *P*>0.9; Fig. 7, right panels). Monarchs without antennae had similar righting behavior to sham-operated monarchs in darkness; all sham-operated monarchs displayed normal righting behavior in all of their trials (Moore's paired test: autumn 2019: *n*=5, *R*′=0.62, 0.5>*P*>0.1; summer 2019: *n*=5, *R*′=0.01, *P*>0.999).

**Fig. 8.**
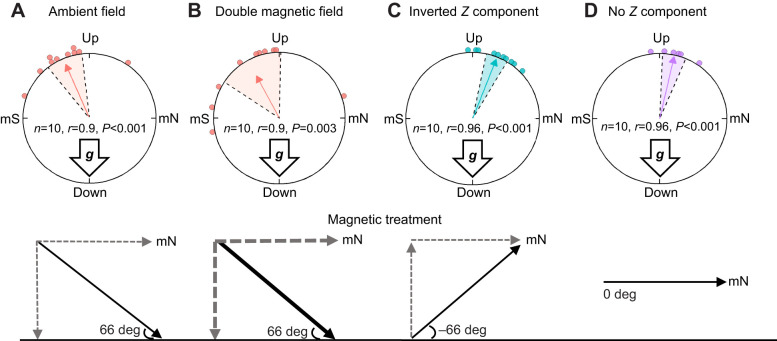
**The effect of magnetic field parameters on vertical orientation during righting behavior.** When the magnetic field was available for orientation, the upwards righting behavior of fall monarchs was deflected towards their perceived autumn migratory direction, which is equatorwards. Monarchs can use the inclination angle of the magnetic field for orientation (see Guerra et al., 2014). (A,B) For trials with the ambient inclination angle, the righting behavior of monarchs was shifted equatorwards (seen by a leftwards shift from the vertical). (C) For trials with an inverted magnetic inclination angle which reversed the physical position of equatorwards and polewards in trials, monarchs shifted their righting behavior in accordance with the flipped inclination angle, which kept them oriented in the proper equatorwards direction. (D) For no inclination angle trials, monarchs oriented upwards and did not significantly shift their body position away from the vertical, as in trials with an inclination angle. For A–D, colored arrows represent the mean orientation of butterflies in each trial group and shaded areas indicate 95% confidence intervals. Directly below each result is a depiction of the artificial magnetic field conditions that we exposed monarchs to during trials. Solid arrows show the total magnetic field, with thickness representing relative strengths. The dashed arrows represent the magnetic field components divided into the horizontal and vertical components of the magnetic field. The inclination angle for each magnetic field is indicated.

When we tested monarchs with gravity and magnetic cues together (with light cues present but non-directional), we similarly found that monarchs moved to be head up and facing upwards, but that the final orientation of monarchs was then shifted equatorwards in relation to the presented magnetic field. This additional equatorwards shift in orientation occurred when monarchs were exposed to either ambient or double-strength magnetic fields (Fig. 8A,B; Table S1). Specifically, our results also directly show that monarchs are directly responding to the inclination angle of the magnetic field after righting themselves, as monarchs shifted in the opposite direction as compared with ambient and double-strength magnetic field conditions when the inclination angle was inverted 180 deg (Fig. 8C; Table S1). These shifted righting orientations were significant across all magnetic field conditions (Table S1), with orientations in ambient and double-strength field conditions similar to each other and both different from those under inverted inclination angle conditions (Hotelling's paired test; ambient versus double strength: *f*=1.0, *P*_FDR_=0.4; ambient versus inverted: *f*=6.3, *P*_FDR_=0.03; double versus inverted: *f*=8.3, *P*_FDR_=0.03). In contrast to conditions in which monarchs were presented with a magnetic field with an inclination angle (ambient field conditions; Fig. 8A–C), monarchs tested without any inclination angle information (0 deg during trials) did not shift their righting response from the upward, vertical position (Hotelling's paired test: ambient versus zero inclination angle, *f*=16.5, *P*=0.01; Fig. 8D; Table S1).

## DISCUSSION

We provide evidence that monarchs sense gravity and can use this environmental sensory cue to facilitate oriented movement (walking and righting behavior) and for overall directionality (sensing upwards). Monarchs only display negative gravitaxis, and can track gravity cues and maintain negative gravitaxis even when the surface that they are walking on is dynamic and physically shifting. Monarchs can sense and use gravity for oriented behavior even in complete darkness, a sensory environment in which there are no directional light or magnetic cues available. Whereas the antennae act as important gravisensors for monarchs, in the absence of antennae, monarchs can still display proper righting behavior under darkness (no light or magnetic cues for upwards directionality), demonstrating that they possess other gravisensors. We found that monarchs can integrate gravity with other environmental sensory cues (light and magnetic cues) for oriented movement and behavior. In the presence of these other sensory cues, gravity was the dominant cue but only when antennae were present. The actual physical relationship that gravity has with these other directional sensory cues also dictates how gravisensation is modulated.

### Gravitaxis and gravisensation

Our results demonstrate that monarchs can use gravity as an environmental cue in at least two different behavioral contexts – for directing upwards movement (negative gravitaxis) and for righting behavior, in which monarchs orient to an upwards (head-up) body position. In monarchs, negative gravitaxis is a threshold response, as it only occurs once monarchs are on a surface that is inclined by at least 60 deg relative to the horizontal. Therefore, not only are monarchs able to sense and use gravity cues but also they possess inclinosensation and are able to detect tilt like other animals when on a surface or substrate (Seidl and Wehner, 2008). Moreover, both gravisensation and inclinosensation in monarchs appear to be dynamic responses, as monarchs can perform and maintain their oriented movement using gravity and tilt cues even when their physical environment (the physical surface that they are on) is actively shifting.

In contrast to that in other species (e.g. fruit flies – Fedele et al., 2014; Toma et al., 2002), gravisensation in monarchs appears to be a light-independent process, as they were able to right themselves in complete darkness in our study. Gravity cues appear to be sufficient for oriented behavior (righting behavior), as monarchs do not have access to other cues in darkness that can provide directional information, such as the vertical component and vector of the magnetic field (Putman et al., 2018). Our results therefore suggest that gravisensation in monarchs is potentially based on a mechanosensory system. We found that the antennae act as important morphological structures for sensing gravity, with intact antennae necessary for upwards orientation and negative gravitaxis. For example, in our experiments in which monarchs had their antennae removed (righting response trials), monarchs no longer shifted their orientation and movement upwards (0 deg) but instead displayed positive phototaxis as they directed their movement towards the position of a directional light source (light source coming from the left, and perpendicular to the vertical plane; Fig. 7). In monarchs, gravity might be sensed by the antennae, as they possess organs that are responsible for gravisensation, as found in other insects (Johnston's organ: fruit flies – Armstrong et al., 2006; Sun et al., 2009; ants – Vowles, 1954; mosquitoes – Boo and Richards, 1975) and are involved in maintaining proper body positioning during movement in other Lepidoptera (Bohm's bristles: flight stabilization in Lepidoptera – Sane et al., 2007). As gravity acts on the antennae, the movement and position of the antennae themselves might enable monarchs to determine upwards and downwards. For instance, the Bohm's bristles are hair plates located at the base of the antennae, which are capable of providing gross localization information about where the antennae are in relation to the head of the butterfly (Sant and Sane, 2018). Additionally, as the antennae are physically separated paired sensory organs that can move independently of one another, monarchs might be comparing the information provided by them for upwards directionality, in a similar manner to the way in which they use independent timing information from each of the antennae during time-compensated sun compass use (Guerra et al., 2012). Given the role of the antennae for gravisensation and their overall morphology (a long, thin flagellum with a much larger club-like section at the distal end), future studies with monarchs might focus on determining the relative torque experienced by the antennae during body orientation positioning, and investigate the electrophysiological responses of neurons downstream from antennal structures. These studies would help elucidate how the brain encodes body orientation and directionality and provide a promising route for understanding how this information is encoded.

Monarchs also appear to possess other gravisensors beyond the antennae that might serve as alternative or back-up gravity sensors. This secondary mechanism for detecting gravity appears to function within a stricter set of sensory conditions, however. For example, in righting response trials in which we removed the antennae, monarchs were able to right themselves to an upwards position (0 deg; Fig. 7, right panels), but only when tested in darkness. This suggests that this other form of gravisensation is likely context dependent, such as functioning at night when monarchs are at rest or quiescent when roosting in trees (and the antennae are relaxed, droop down and are at rest close to the body) or is a gravisensation mechanism secondary to phototaxis. Possible candidates for this secondary mechanism are the campaniform sensilla and hair plates located at the thorax–coxa joint (Bässler, 1971). Campaniform sensilla are specialized mechanoreceptors imbedded in the appendages of insects capable of determining the forces experienced by these appendages (Dey et al., 1995), and the hair plates are important for proprioception (Tuthill and Wilson, 2016). The forces sensed by the campaniform sensilla and the limb position monitored by the hair plates might provide directional information derived from gravity cues in a similar mechanical manner to the antennae.

Why are sensing gravity, negative gravitaxis and righting behavior adaptive for monarchs? One possible ecological explanation is that gravity cues play a key role for flight take-off. Righting behavior (whether righting themselves or maintaining a head-up position) can first help monarchs be in a more advantageous physical position, across a variety of contexts, but specifically for flight readiness. An initial hurdle in flight kinematics is the ability to generate enough acceleration to initiate flight. Butterflies (Bimbard et al., 2013), other insects (Burrows et al., 2019) and birds (Heppner and Anderson, 1985) have been shown to overcome this first hurdle by initially leaping into the air followed by wing flapping. It is possible that monarchs are looking to increase their physical elevation prior to this initial leap. Both migratory and non-migratory monarchs can utilize this technique for initiating flight by jumping from a higher location. In support of this possible function, we observed that some monarchs had initiated flight upon successfully reaching the top of the mesh tube in our trials. Another ecological explanation for negative gravitaxis could be related to thermoregulation. Monarchs have been observed to utilize the sun to warm up their flight muscles prior to flying (McCord and Davis, 2010). By walking upward on a substrate, monarchs can reach higher locations with less cover to bask in the sun under natural conditions. This behavior would be especially helpful during cooler months, such as late autumn and early spring, when monarchs undertake their migratory journey, and during early mornings when temperatures are typically lower. Finally, monarchs can use gravity as a reliable cue for directional information and oriented movement as it is an ever-present cue in the environment throughout the day (both day and night), can be used irrespective of current weather conditions (unlike sun visual cues that can be occluded during overcast conditions), and does not require sensory input from other modalities (unlike light-dependent magnetosensation – Guerra et al., 2014; Wan et al., 2021).

### Multimodal sensory integration

Our results demonstrate that monarchs can sense and integrate cues from different sensory modalities together to produce oriented movement. In all our experiments examining sensory integration, the response to gravity cues was dominant over the responses to directional light or magnetic field cues. This was demonstrated as monarchs only moved with negative gravitaxis (gravisensation tube assay), and always righted themselves upwards (righting response assay, head-down starting position) or maintained a head-up position (righting response assay, head-up starting position) if their antennae were intact and able to fully sense gravity during multimodal cue situations. We found that the behavioral responses during multimodal sensory contexts varied with the physical positions of directional multimodal cues relative to the animal.

When directional light cues were presented from below the tube in gravitaxis trials (tube in a vertical position), monarchs readily performed negative gravitaxis with the light cues having no effect on movement. Similarly, directional light cues also had no effect when presented from behind monarchs during righting response trials, with monarch righting behavior unaffected by these light cues. Additionally, although directional light cues (light from either the left or right of the monarchs) did skew monarch orientation towards the light source in righting response trials (10–15 deg shift towards the light source relative to the position of 0 deg; Fig. 6), monarchs always righted themselves to a head-up, upright position that was distinct from the positive phototactic response that we observed when monarchs had no antennae in righting response trials. This pattern of results from our experiments therefore suggests a hierarchical structure between gravity and directional light cues. If gravity and directional light cues were weighted equally by monarchs, we would have expected monarchs to have shifted 45 deg relative to the vertical when the light was on either side in righting response trials, or a random orientation when the two cues were in direct conflict during trials when light was from below. Instead, our results show that gravity cues can be dominant over light cues during orientation behavior in monarchs, with gravity cues inhibiting positive phototaxis to directional light cues. Such a hierarchical structure between multimodal sensory cues has been previously observed in fall migratory monarchs within the context of the cues used for oriented southwards flight (Guerra, 2020). Here, the predominant compass mechanism monarchs use to maintain southwards flight is a time-compensated sun compass, with monarchs using the sun as a directional visual cue. When the sun's position is occluded during overcast sky conditions preventing the use of directional visual cues, monarchs then rely on a backup magnetic compass for flying southwards, with the compass tuned to the inclination angle of the magnetic field (equatorwards flight). Additionally, directional light cues can modulate the expression of gravitaxis and this modulating effect is dependent on the relative localizable position of light relative to individuals. A similar relationship between gravity and light cues has also been observed in other taxa (Daiker et al., 2011; Kwon et al., 2016). For instance, in *Euglena gracilis*, responses can be gravity biased, with gravity-based responses modulated by light, depending on the environmental conditions faced by individuals and the intensity of cues (Daiker et al., 2011; Ozasa et al., 2019).

We observed a similar pattern between multimodal cues when monarchs had access to both gravity and magnetic field cues together during our righting response trials under diffuse light conditions that provided the necessary light input to activate magnetosensation. Magnetic field cues also shifted the orientation of fall migratory monarchs during righting behavior. Here, the head-up, upright orientation of the monarch righting response was shifted equatorwards, the expected orientation of monarchs in these trials, as it is the orientation consistent with the monarch's autumn migratory direction and what was observed in previous studies (flight simulator trials; Guerra et al., 2014), when tested under our artificially generated magnetic fields (ambient and double-strength magnetic fields; Fig. 5). This shift was similarly performed, but in the opposite direction, when we reversed the inclination angle of the magnetic field in trials (Fig. 5) and no shift occurred when monarchs were presented with no discernible inclination angle (0 deg inclination; Fig. 8D; Table S1). Both responses in righting behavior are like those observed when fall monarch flight orientation was tested in flight simulator trials (Guerra et al., 2014), with monarchs reversing their flight orientation with a reversed inclination angle, and flying non-directionally when no inclination angle (0 deg) was present. As monarchs right themselves normally in complete darkness, the shift in upright orientation that we observed when magnetic cues were available suggests a combinatorial effect, rather than a hierarchical relationship, when these two cues are presented together. Gravity and magnetic cues appear to not conflict or inhibit each other, which was shown when monarchs still oriented with a normal upwards, head-up position when the vertical component and inclination angle were inverted in righting response trials. Monarchs simply shifted their orientation relative to vertical (0 deg) with a similar magnitude but in the opposite direction to remain consistent with equatorwards directionality (Fig. 8C).

The response by monarchs to conditions with no discernible inclination angle is consistent with that of other migratory species that use the inclination angle of the magnetic field for orientation. For example, in experiments with white-crowned sparrows, an inclination angle near zero made it difficult for these birds to orient using their inclination angle magnetic compass (Åkesson et al., 2001). It is likely difficult for monarchs to determine equatorward versus poleward without a discernible inclination angle. As gravity cues were still present, monarchs therefore oriented to the vertical, upwards position as expected.

The responses of monarchs when gravity and magnetic field cues are found together share similarities and differences with the responses of other animals to these two types of sensory cues. For example, as we observed in monarchs, the movement (upwards) of young Chinook salmon (Putman et al., 2018) did not differ between ambient and intensified magnetic field conditions. Additionally, as in monarchs, the movement of these fish was affected when the inclination angle was reversed. But unlike monarchs, which moved with the same magnitude despite the reversal, the upwards movement of fish was significantly reduced when the vertical component of the magnetic field was inverted (from downwards to upwards) and in direct conflict with the downwards directional vector of gravity.

### Antennae as multimodal sensors

Our results further demonstrate that the antennae of monarchs act as peripheral multimodal sensory structures (Guerra and Reppert, 2015). In addition to playing a role in sensing gravity, the antennae have previously been shown to be sensitive to light cues (light-entrained antennal clocks for proper time-compensated sun compass use – Merlin et al., 2009; Guerra et al., 2012), are the location of wavelength-dependent, light-activated magnetosensors relevant for inclination-based magnetic compass use (Guerra et al., 2014), and are involved in both contact (Haribal and Renwick, 1998) and volatile (Bergström et al., 1994) chemoreception. Given their location, anatomy and ability to move (whether by the monarch or by external forces), the antennae of monarchs might also play a role in sensing other cues such as temperature, barometric pressure, wind or acoustic cues as in other insects (Guerra and Reppert, 2015). How information from these different sensory cues is sent downstream from the antennae to the central complex region of the brain (Heinze et al., 2013; Heinze and Homberg, 2007; Heinze and Reppert, 2011, 2012) for producing specific behaviors remains unknown. Previous work, however, has demonstrated that the paired antennae of monarchs can function independently of each other. During the sensing of environmental cues, there appears to be no crosstalk between antennae, with each antenna providing separate and distinct information derived from sensory conditions that are then sent downstream (e.g. independent circadian clock information – Guerra et al., 2012).

### Further insights

The behavioral responses of monarchs to magnetic cues in our study provides further evidence that monarchs are capable of magnetosensation. Our results also demonstrate that monarchs can derive directional information from the magnetic field, as they respond to the inclination angle of the magnetic field to guide oriented movement and body positioning (Guerra et al., 2014; Wan et al., 2021). In addition, as we showed that monarchs are capable of gravisensation and respond to both gravity and magnetic field cues when together, we now have evidence for a mechanism (as suggested in Wiltschko and Wiltschko, 1972) for how animals can sense up and down to properly interpret the inclination angle of the Earth's magnetic field in 3D space when using inclination angle for directionality during movement. Such a mechanism of using gravity for sensing up and down might also be used by other species that similarly use the inclination angle of the geomagnetic field for oriented movement, such as birds, sea turtles and fish (reviewed in Lohmann et al., 2007). The ability of monarchs to sense and move with respect to gravity provides them with this key piece of information during magnetic compass use; that is, the use of gravity and the direction of the gravitational vector as a key reference point for interpreting inclination angle (Wiltschko and Wiltschko, 1972).

Finally, the oriented responses of monarchs in righting response trials during which gravity and magnetic field cues are present together are consistent with the migratory restlessness observed in birds. Specifically, the shifted head-up, upwards righting response of monarchs equatorwards is like the behavior of migratory birds experiencing migratory restlessness (Zugunruhe). For example, migratory birds will move and orient in the proper seasonal migratory direction when tested in behavioral assays (Emlen funnels), movement that is independent of actual migratory flight behavior (Bianco et al., 2016; Emlen and Emlen, 1966; Wiltschko and Wiltschko, 1972). For monarchs, the preference for upright, equatorwards-oriented body positioning as seen in our righting behavior assay might be an additional trait that forms part of the monarch migratory syndrome.

## Supplementary Material

10.1242/jexbio.245451_sup1 Supplementary information
