# Supplementary material for: Gravisensation and modulation of gravitactic responses by other sensory cues in the monarch butterfly (Danaus plexippus)
Source: J Exp Biol. 2023 Nov 7;226(21):jeb245451. doi: 10.1242/jeb.245451 (PMC10651108; doi:10.1242/jeb.245451)
Supplement: Supplementary information [file jexbio-226-245451-s1.pdf]

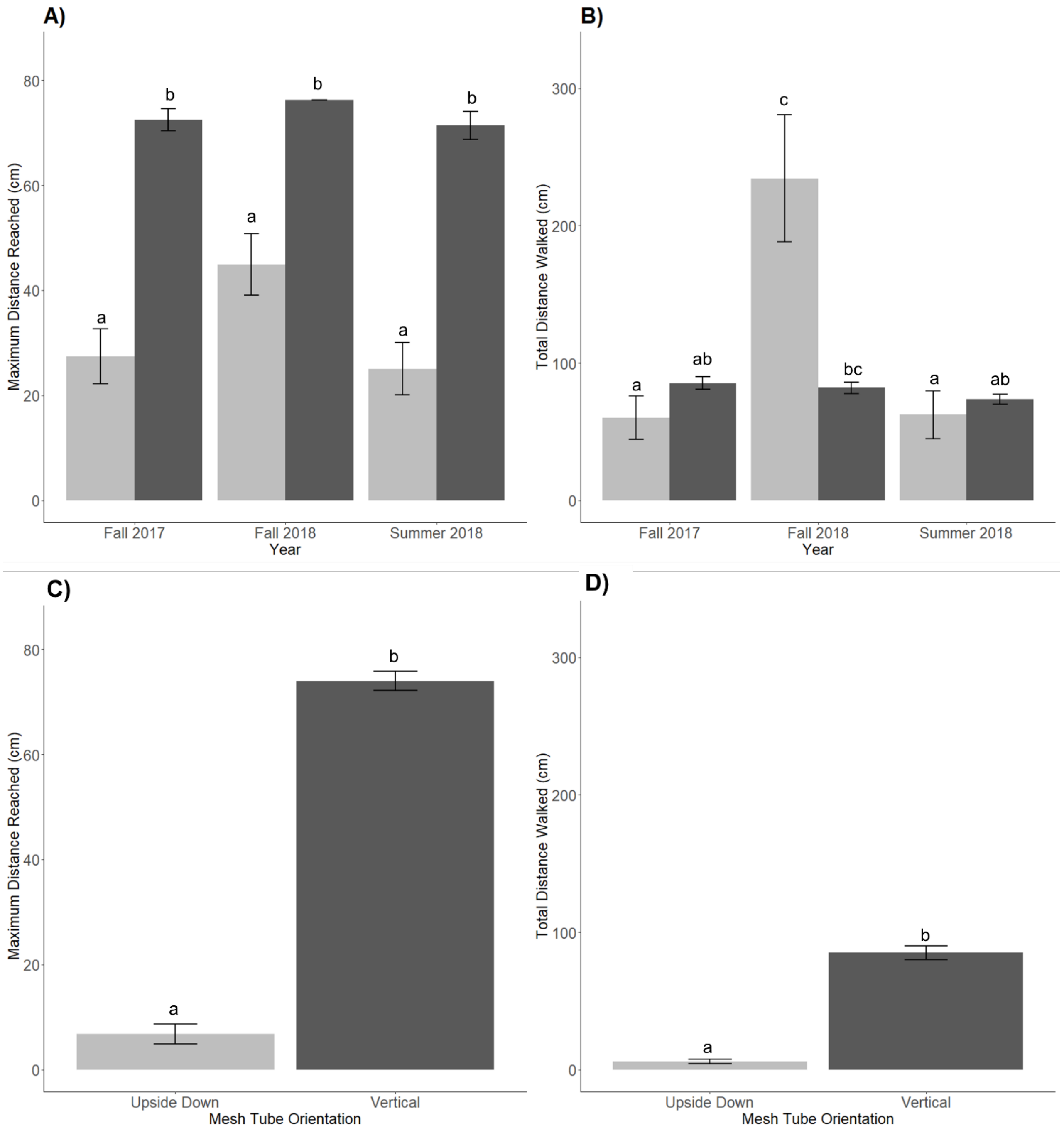

Fig. S1. Post-hoc comparisons for negative gravitaxis and positive gravitaxis assays. A) The mean maximum distance reached in each tube orientation trial for negative gravitaxis assay trials. **B)** The mean total distance walked in each tube orientation trial for negative gravitaxis assay trials. For both A-B, light gray bars represent trials with the mesh tube in the horizontal orientation and dark

Journal of Experimental Biology • Supplementary information

gray bars represent the trials with the tube in the vertical orientation (Fall 2017: n = 20; Fall 2018: n = 21; Summer 2018: n = 21). **C)** The mean maximum distance reached in each tube orientation trial for positive gravitaxis assay trials (n = 21). **D)** The mean total distance walked in each tube orientation trial for positive gravitaxis assay trials (n = 21). For all panels, error bars represent the standard error of the mean. Within each panel, bars that do not share a letter are significantly different from each other (Tukey's post-hoc p-value adjustment method for multiple comparisons; $p < 0.05$).

Journal of Experimental Biology • Supplementary information

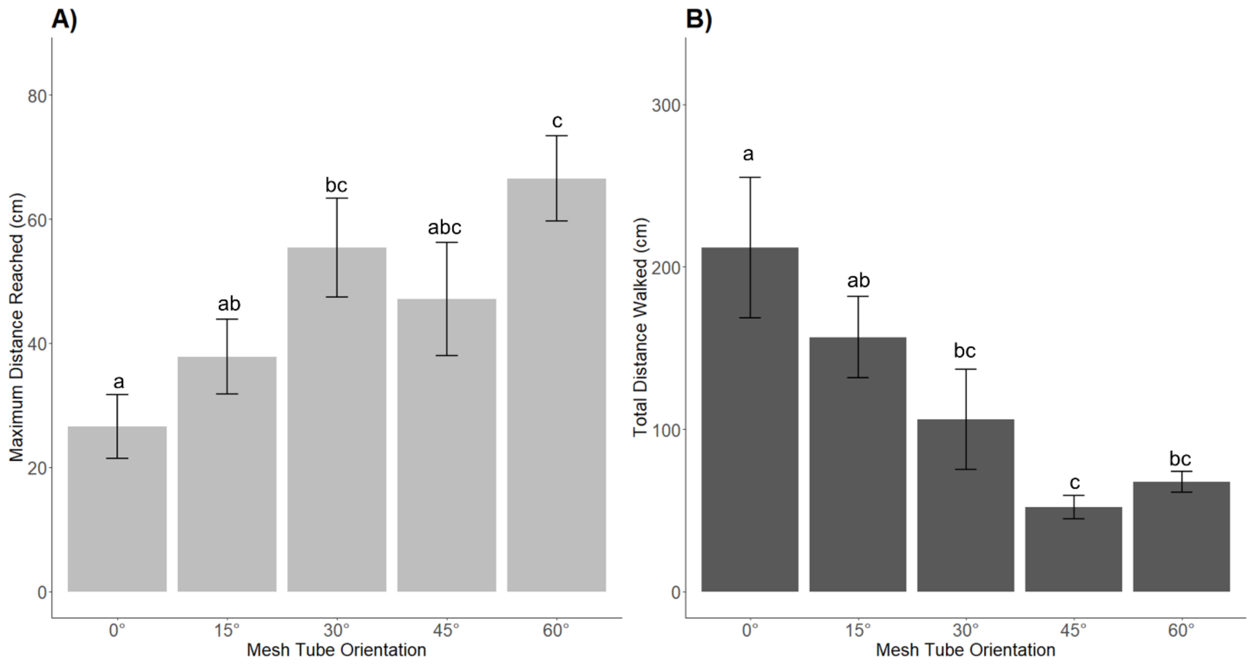

Fig. S2. Post-hoc comparisons for inclination trials. A) The mean maximum distance reached in each tube orientation trial (n = 10 monarchs each tested across all tube orientations). **B)** The mean total distance walked in each tube orientation trial (n = 10 monarchs each tested across all tube orientations). For both panels, error bars represent the standard error of the mean. Within each panel, bars that do not share a letter are significantly different from each other (Tukey's post-hoc p-value adjustment method for multiple comparisons; $p < 0.05$).

Journal of Experimental Biology • Supplementary information

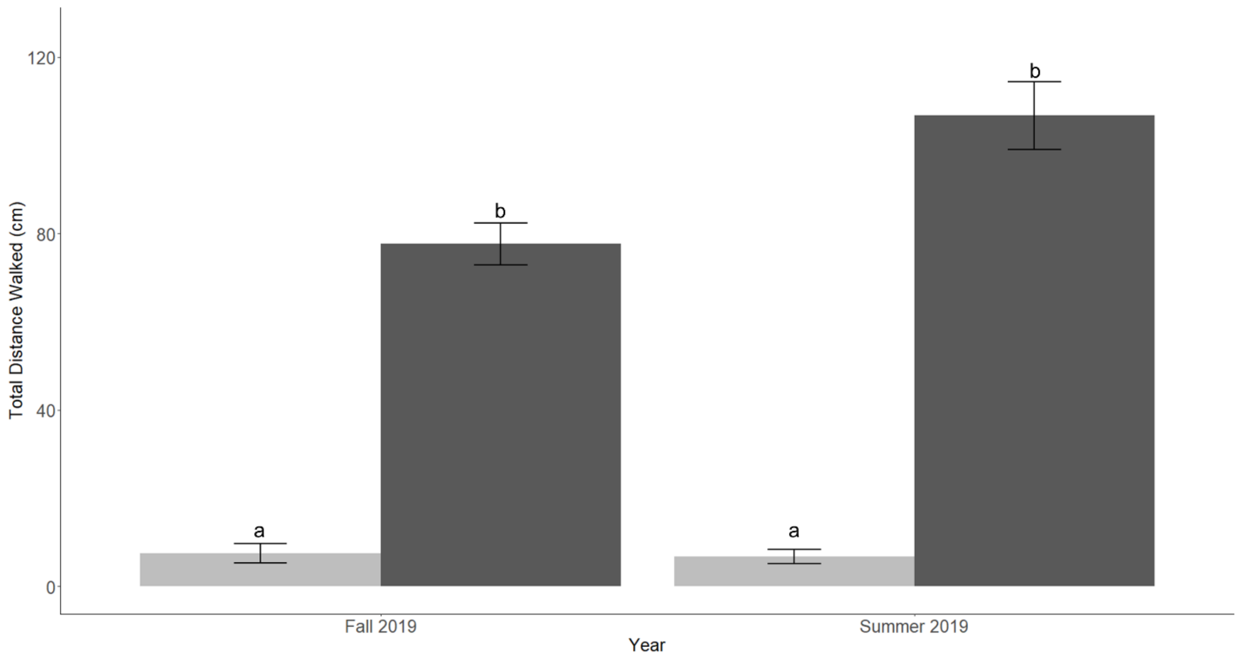

Fig. S3. Post-hoc comparisons of dynamic tube trials. The mean distance traveled either downward (total negative distance; light gray bars) or upward (total positive distance; dark gray bars) for fall 2019 (left; n = 10) and summer 2019 (right; n = 10) monarchs. Error bars represent the standard error of the mean. Bars that do not share a letter are significantly different from each other (Tukey's post-hoc p-value adjustment method for multiple comparisons; $p < 0.05$).

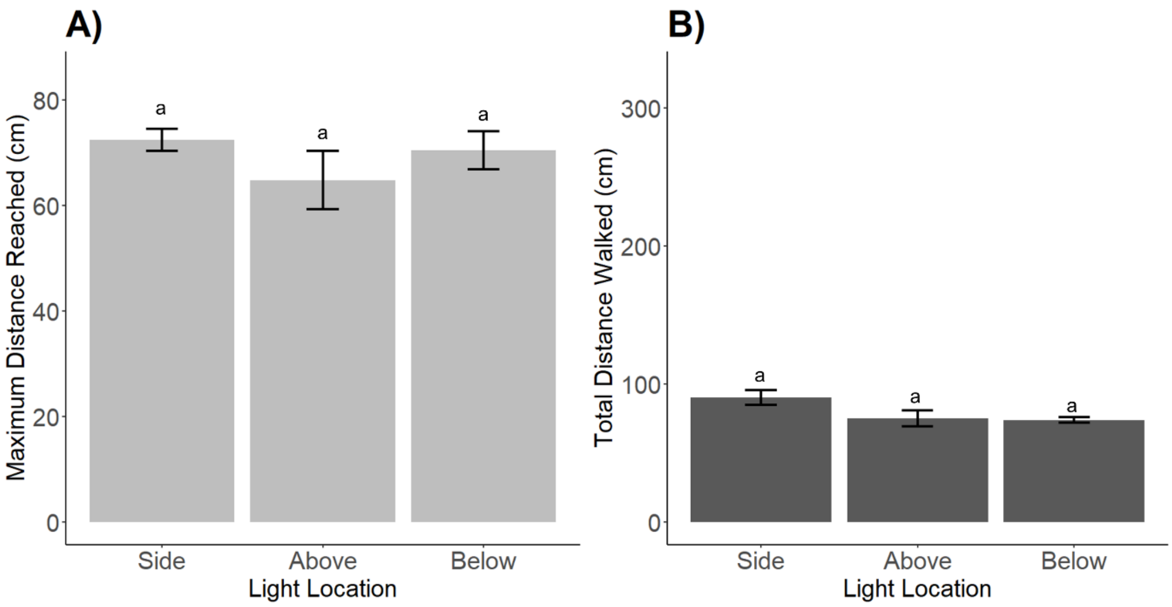

Fig. S4. Post-hoc comparisons of phototaxis assays. A) The mean maximum distance reached in each tube orientation trial (n = 20 for side group which is the baseline treatment – first trial; n = 10 for each of above and below groups – second trial). **B)** The mean total distance walked in each tube orientation trial. For both plots, error bars represent the standard error of the mean. Within each panel, bars that do not share a letter are significantly different from each other (Tukey's post-hoc p-value adjustment method for multiple comparisons; $p < 0.05$).

Journal of Experimental Biology • Supplementary information

Table S1. Results of Rayleigh's tests for parametric righting response orientation assay data.

Experimental condition	Trial		n	r	mean orientation (°)	95% confidence interval (°)	p-value
Orientation with Gravity alone: Fall 2019	Light Off:	Head Up	15	0.898	358.84	4.56 - 351.57	<0.0001
		Head Down	15	0.872	359.84	15.26 - 344.98	<0.0001
	Light on Left:	Head Down	15	0.898	317.85	331.18 - 309.33	<0.0001
Orientation with Gravity alone: Summer 2019	Light Off:	Head Up	15	0.975	356.48	4.9 - 347.72	<0.0001
		Head Down	15	0.855	358.7	15.23 - 346.3	<0.0001
	Light on Left:	Head Down	15	0.91	325.69	315.31 - 339.3	<0.0001
Gravity and Light Integration: Fall 2018	Light on left:	Head Up	21	0.962	350.76	342-359.82	<0.0001
		Head Down	21	0.886	339.82	323.29-355.41	<0.0001
	Light on Right:	Head Up	21	0.946	19.29	7.3-27.65	<0.0001
		Head Down	21	0.938	29.03	16.47-39.08	<0.0001
	Light Underneath:	Head Up	21	0.932	5.7	353.11-17.47	<0.0001
		Head Down	21	0.886	8.13	351.84-23.82	<0.0001

Gravity and Light behind: Fall 2018	Light Behind:	Head Up	10	0.991	5.21	18.75 - 359.77	<0.0001
		Head Down	10	0.964	7.97	0.24-10.03	<0.0001
	Light on Right:	Head Down	10	0.907	39.19	23.9-55.59	<0.0001
Gravity and Light Integration: Summer 2018	Light on left:	Head Up	21	0.952	336.58	328.92 - 344.3	<0.0001
		Head Down	21	0.851	330.57	319.8 - 344.28	<0.0001
	Light on Right:	Head Up	21	0.98	30.54	25.68 - 35.45	<0.0001
		Head Down	21	0.912	29.98	19.8 - 40.77	<0.0001
	Light Underneath:	Head Up	21	0.936	4.64	13.42 - 355.51	<0.0001
		Head Down	21	0.828	350.6	6.97 - 336.23	<0.0001
Gravity and Magnetic Integration: Fall 2019	Ambient Magnetic Field:	Head Down	10	0.904	336.86	353.46 - 321.58	<0.0001
	Double Magnetic Field:	Head down	10	0.73	331.73	1.31 - 303.3	0.0026
	Inverted magnetic Field:	Head Down	10	0.965	21.57	30.98 - 11.87	<0.0001

No Inclination Angle: Fall 2019	Ambient Magnetic Field:	Head Down	6	0.919	332.49	313.89-351.02	0.0018
	Zero Magnetic Field:	Head Down	6	0.975	12.64	2.49-22.95	0.00003

Journal of Experimental Biology • Supplementary information

Table S2. Results of V-test (Modified Rayleigh's Test) for non-parametric righting response orientation assay data.

Experimental Conditions	Trial	Predicted *a priori* mean orientation (°)	n	r	Observed mean orientation (°)	95% confidence interval (°)	p-value
Mechanism for Gravity Orientation: Fall 2019	Pre-Sham	0	5	0.839	341.63	7.5 - 318.83	0.0022
	Pre-Surgery	0	5	0.711	320.03	297.91 - 339.46	0.01
	Post-Sham	0	5	0.832	330.4	315.9 - 345.16	0.0024
	Post-Surgery	0	5	0.247	284.69	271.55 - 295.91	0.23
	Post-Surgery	270	5	0.941	284.69	271.55 - 295.91	0.0003
Mechanism for Gravity Orientation: Summer 2019	Pre-Sham	0	5	0.623	309.33	301.37 - 319.39	0.023
	Pre-Surgery	0	5	0.764	322.85	312.57 - 340.37	0.0057
	Post-Sham	0	5	0.833	329.87	315.84 - 342.82	0.0023
	Post-Surgery	0	5	0.0023	269.81	248.93 - 292.3	0.5
	Post-Surgery	270	5	0.91	269.81	248.93 - 292.3	0.0007

Journal of Experimental Biology • Supplementary information

Orientation Without Light Cues or Primary Gravity Cue: Fall 2019	Pre-Sham	0	5	0.921	343.09	327.51 - 353.77	0.0005
	Pre-Surgery	0	5	0.938	1.97	18.97 - 344.58	0.0003
	Post-Sham	0	5	0.961	357.1	10.09 - 341.56	0.0002
	Post-Surgery	0	5	0.632	3.49	40.53 - 299.15	0.021
Orientation Without Light Cues or Primary Gravity Cue: Summer 2019	Pre-Sham	0	5	0.863	1.5	26.99 - 331.21	0.0015
	Pre-Surgery	0	5	0.858	0.4	27.48 - 332.06	0.016
	Post-Sham	0	5	0.864	0.67	27.41 - 334.64	0.0015
	Post-Surgery	0	5	0.934	353.14	13.55 - 338.14	0.005

Journal of Experimental Biology • Supplementary information
